# Anomalous Coulomb-Enhanced Charge Transport in Triangular Triple-Quantum-Dot Systems

**DOI:** 10.3390/e28040441

**Published:** 2026-04-14

**Authors:** Shuo Dong, Junqing Li, Jianhua Wei

**Affiliations:** School of Physics, The Renmin University of China, Beijing 100876, China; dongshuo@ruc.edu.cn (S.D.); lijunqing@ruc.edu.cn (J.L.)

**Keywords:** berry phase, quantum dots, quantum computation

## Abstract

Electron correlation and quantum interference are pivotal in mesoscopic transport. We theoretically study the nonequilibrium transport dynamics of a triangular triple-quantum-dot (TTQD) molecule connected to fermionic reservoirs using the exact hierarchical equations of motion (HEOM) formalism. We demonstrate a counterintuitive transport signature in which the stationary current is significantly enhanced by increasing *U*, a behavior distinct from the suppression typically observed in linear quantum dot arrays. By analyzing the evolution of spectral functions, we attribute this enhancement to the interplay between Coulomb-interaction-induced energy shifts and quantum interference effects specific to the triangular topology. We also explore how the circulation of chiral currents and electrode coupling strength modulate these interaction effects. Finally, we present a three-dimensional map of the transport current as a function of inter-dot tunneling (*t*) and Coulomb interaction (*U*), illustrating their combined effect on the current magnitude and its applications.

## 1. Introduction

Quantum dots (QDs), often described as “artificial atoms,” have become one of the most widely studied platforms in condensed matter physics and nanoscience. These nanoscale semiconductor structures confine electrons in all three spatial dimensions, yielding discrete, atom-like energy levels that are tunable through external gate voltages and magnetic fields [1,2]. This tunability has made quantum dots a natural setting for studying fundamental quantum phenomena in controllable mesoscopic systems and for developing quantum technologies. The foundation of quantum dot physics was established through the experimental observation of single-electron charging effects. Meirav, Kastner, and Wind demonstrated periodic conductance resonances in GaAs nanostructures, providing the first clear evidence of Coulomb blockade in quantum dots [3]. These pioneering experiments showed that when a quantum dot is weakly coupled to electron reservoirs, the addition energy required to place each successive electron on the dot produces a distinctive pattern of conductance peaks as a function of gate voltage. Kouwenhoven and colleagues further advanced the understanding of discrete energy levels and Coulomb charging effects, establishing quantum dots as true artificial atoms with a shell structure [4,5].

Central to quantum dot physics is the on-site Coulomb repulsion energy *U*, which characterizes the electrostatic cost of placing two electrons on the same dot. The theoretical framework for understanding *U* was established by Beenakker, who provided an early treatment of Coulomb blockade oscillations in quantum dot conductance [6]. In the Hubbard model description of quantum dots [7], *U* competes directly with the kinetic energy scale set by the interdot tunneling amplitude *t*. When U≪t, electrons delocalize freely and transport is relatively unimpeded. As *U* increases toward and beyond *t*, charge fluctuations become energetically costly, electrons tend to localize on individual dots, and the system enters a strongly correlated regime in which conventional single-particle descriptions break down. This competition between *U* and *t* is a genuine control parameter that drives the system through qualitatively distinct physical regimes, from weakly interacting Fermi-liquid behavior to strongly correlated Mott-like insulating states. How *U* shapes transport properties is thus a central issue in quantum dot physics. Loss and DiVincenzo’s seminal proposal established electron spins confined in quantum dots as viable qubits, highlighting their long coherence times and gate-voltage controllability as key advantages for scalable quantum computation [8]. Following this proposal, subsequent experiments demonstrated precise control over individual and coupled spin qubits [9,10,11,12]. These developments established quantum dots as leading candidates for solid-state quantum computing, and simultaneously underscored the importance of understanding Coulomb interactions, since *U* directly governs the exchange splitting between singlet and triplet spin states that underpins spin qubit operation.

Linear quantum dot arrays, from double-quantum-dot (DQD) to triple-quantum-dot (TQD) systems, have been extensively studied as building blocks for quantum computation and as platforms for exploring interdot coupling, tunneling dynamics, and multi-qubit operations. For DQDs, Van der Wiel et al. provided a comprehensive review of electron transport, discussing the interplay among interdot tunneling, lead coupling, and Coulomb interactions [13]; coherent charge oscillations were demonstrated by Hayashi et al. [14], and the Pauli spin blockade was observed by Ono et al. and Johnson et al. [15,16]. Extending to TQDs, Schröer et al. realized electrostatically defined serial devices with controllable electron numbers [17], while Gaudreau and colleagues fabricated tunable few-electron TQDs and observed coherent three-dot coupling [18,19]; the transport theory was further developed by Rogge and Haug, who highlighted quantum interference between different tunneling pathways [20,21], and by Michaelis et al. using rate equations [22]. Across both DQD and linear TQD configurations, the role of the on-site Coulomb interaction *U* is qualitatively consistent: stronger repulsion raises the addition energy, widens the Coulomb blockade valleys, localizes electrons on individual dots, and suppresses sequential tunneling, reinforcing the conventional expectation that *U* impedes rather than facilitates current flow.

Moving beyond linear quantum dots, triangular triple-quantum-dot (TTQD) systems are particularly intriguing because they break linear symmetry and introduce geometric frustration, potentially giving rise to novel quantum phenomena. The threefold rotational symmetry of triangQDs creates fundamentally different interference conditions compared to linear arrangements. Korkusinski et al. investigated the electronic properties of triangular lateral quantum dot molecules and introduced “topological Hund’s rules” to describe state filling [23], showing that geometry alone can qualitatively alter the energy spectrum and charge configurations. Experimentally, Granger et al. mapped out the three-dimensional transport diagram of a TQD, revealing complex charge stability regions in multi-parameter space [24]. Seo et al. demonstrated charge frustration effects in a triangular TQD arising from the geometric arrangement [25], showing that the triangular geometry leads to degenerate charge configurations and non-trivial ground states that differ qualitatively from linear arrangements. On the theoretical side, Žitko, Bonča, and Pruschke investigated quantum phase transitions in TQDs using numerical renormalization group methods, finding that the interplay between Kondo screening and geometric structure can lead to complex phase diagrams [26].

Recent advances in quantum-dot systems and nanoscale transport have also highlighted the need for analytical and conceptually transparent descriptions of coupled electronic and vibrational dynamics. Prat V.P. et al. obtained perturbative solutions for the three-state Rabi model and the linearized triple-quantum-dot shuttle, showing that perturbation theory is restricted in the former case but, with degeneracy properly treated, can reproduce anticrossings in the latter [27]. In open quantum-dot systems, Yiyang Li et al. showed that a non-Hermitian formulation naturally captures exceptional points and relates them to chiral transport and transmission resonances [28]. For coupled quantum-dot molecules, Nagwa et al. found that intrinsic decoherence suppresses entanglement and coherence, whereas Coulomb interactions tend to stabilize these quantum correlations [29]. At the same time, defect dynamics in layered materials has been addressed in Daniel et al.’s work [30], where a minimal model for chalcogen vacancies in transition-metal-dichalcogenide graphene heterobilayers was developed and used to clarify the roles of virtual charge fluctuations, symmetry, and spin-orbit coupling in charge relaxation.

Yet, despite this extensive body of work, a basic question about transport in triangular TQDs has not been systematically examined. For single dots and linear multi-dot systems, it is well established that increasing the charge fluctuations by making them energetically costly drives the system toward electron localization [13,31,32]. However, this picture was developed primarily for systems with simple, effectively one-dimensional tunneling topologies, in which a single dominant transport pathway exists, and *U* acts uniformly to block it. The triangular TQD geometry violates this assumption in a fundamental way. With three dots arranged in a closed loop, electrons have access to multiple interfering paths whose phase relationships are not fixed by geometry alone but are renormalized by interactions. The closed-loop structure introduces a topological character analogous to that of Aharonov–Bohm physics, even in the absence of external magnetic fields. Geometric frustration prevents the simultaneous optimization of all pairwise interactions, potentially stabilizing unusual ground states. The absence of left-right mirror symmetry distinguishes the triangular configuration from all linear arrangements and removes constraints that would otherwise pin the interference conditions. These features together raise a question that has not been addressed: does increasing *U* suppress conductance in a triangular TQD as in simries, or can the interplay between strong Coulomb correlations and closed-loop topology produce qualitatively different behavior?

Addressing this question matters for both fundamental and practical reasons. Identifying a regime in which strong correlations enhance rather than suppress transport would pointopic physics, one that expands our understanding of how electron-electron interactions operate in confined geometries. At the same time, quantum dot arrays are actively being developed for quantum simulation, computation, and sensing, and triangular geometries are increasingly explored as building blocks for two-dimensional quantum dot arrays. If *U* can enhance conductance in such geometries, this opens a design principle for interaction-controlled quantum devices. For TQD-based qubits specifically, understanding how *U* affects transport and coherence across a wide parameter range is essential for device optimization, since qubit operation requires precise control over both charge and spin degrees of freedom that *U* directly governs.

In this work, we investigate the transport properties of a triangular TQD across a wide range of Coulomb interaction strengths using the hierarchical equations of motion (HEOM) method. The hierarchical equations of motion (HEOM) approach examines quantum dot properties in equilibrium and nonequilibrium states through the reduced density operator, which possesses a universal formalism applicable to arbitrary system Hamiltonians. The transport problem considered here lies in a regime where strong local interaction, coherent interdot tunneling, and finite bias must be treated simultaneously. While the numerical renormalization group (NRG) is highly successful for equilibrium strong-correlation physics, its extension to finite-bias transport in multiquantum-dot systems is considerably more challenging. Nonequilibrium Green’s function (NEGF) methods are well-suited for transport under bias, but their treatment of many-body interactions typically relies on approximations that may become unreliable in the intermediate-to-strong correlation regime. We therefore employ the hierarchical equations of motion (HEOM), which provide a nonperturbative and numerically controlled description of nonequilibrium open quantum systems. HEOM is particularly suitable here because it allows us to resolve both the steady-state current and the interaction-induced spectral redistribution.

Our central finding is that increasing the on-site Coulomb repulsion *U* enhances conductance through the system, in stark contrast to the conventional expectation that stronger interactions suppress current flow. This effect is intrinsically tied to the closed-loop triangular geometry and does not occur in linear TQD configurations, confirming that the interplay between Coulomb-interaction-induced energy shifts and quantum interference effects unique to the triangular topology drives the phenomenon. Our results demonstrate a mechanism for harnessing strong correlations constructively, with potential implications for interaction-controlled quantum devices and for a broader understanding of correlated transport in geometrically frustrated systems.

## 2. Theory and Methods

The theoretical treatment of transport through interacting quantum dot systems in the nonequilibrium regime requires sophisticated many-body techniques.

**Figure 1:**
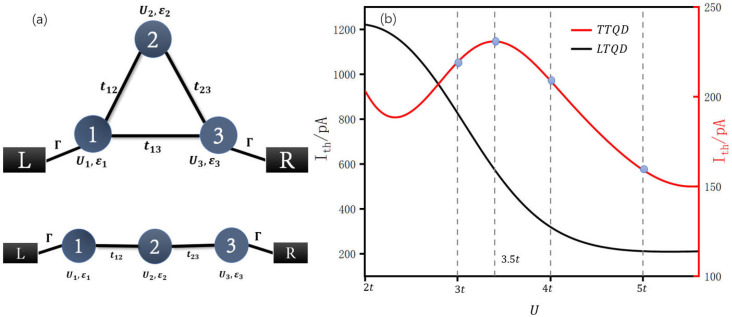
(**a**) Schematic diagrams of a triangular triple quantum dots (TTQDs) and a linear triple quantum dots (LTQDs). Quantum dot 1 and 3 are connected to the left and right electrodes. (**b**) Comparison of the transport current as a function of the on-site Coulomb interaction *U* for the TTQDs and LTQDs configurations. The filled markers on the TTQD curve denote the specific values of *U* at which the spectral function A(ω) has been explicitly evaluated, with all other parameters held identical and the inter-dot hopping fixed at *t* = 0.25 meV. The other parameters are set as follows: $k_{B}T$ = 0.1 meV, Γ = 0.025 meV, *V* = 0.1 meV.

The system consists of three quantum dots arranged in a triangular geometry, as shown in Figure 1a, where all on-site energies and intra-dot Coulomb interactions are identical, and the inter-dot hopping amplitude *t* is uniform across all three links. Two of the three quantum dots (labeled dot 1 and dot 3) are each coupled to a separate fermionic electron reservoir, and a small bias voltage *V* is applied between the two reservoirs to drive a transport current through the system. Another quantum dot (dot 2) is not directly coupled to any reservoir but participates in transport indirectly through its hybridization with dots 1 and 3. Throughout the calculation, electron-hole symmetry is maintained by setting the on-site energy $\varepsilon_{d}=-U/2$, where *U* is the Coulomb interaction strength.

The Anderson impurity model provides the theoretical framework for studying this system. In the multi-impurity generalization, each quantum dot is characterized by an on-site energy level, an intra-dot Coulomb repulsion *U*, and hybridization both with neighboring dots through hopping *t* and with the continuum of conduction electrons in the attached reservoirs. The Hamiltonian of the full system can be written as

(1) $$H=H_{\mathrm{dots}}+H_{\mathrm{leads}}+H_{\mathrm{hyb}},$$

where $H_{\mathrm{dots}}$ contains the on-site energies, the Coulomb repulsion, and the inter-dot hopping among all three dots:

(2) $$H_{\mathrm{dots}}=\sum_{i\mu }\varepsilon_{i}{\hat{d}}_{i\mu }^{†}{\hat{d}}_{i\mu }+U\sum_{i}{\hat{n}}_{i↑}{\hat{n}}_{i↓}+\sum_{i\neq j\mu }t_{ij}{\hat{d}}_{i\mu }^{†}{\hat{d}}_{j\mu }.$$

The ${\hat{d}}_{i\mu }^{†}\left({\hat{d}}_{i\mu }\right)$ in the above formula is a creation (annihilation) operator for an electron with μ spin on the i-th dot. $H_{\mathrm{leads}}$ describes the two noninteracting electron reservoirs:

(3) $$H_{\mathrm{leads}}=\sum_{\alpha k\mu }\varepsilon_{\alpha k}{\hat{c}}_{\alpha k\mu }^{†}{\hat{c}}_{\alpha k\mu },$$

${\hat{c}}_{\alpha k\mu }^{†}\left({\hat{c}}_{\alpha k\mu }\right)$ is a creation (annihilation) operator for an electron of lead α on the *k*-th state, and $\epsilon_{k\alpha }$ is the energy of an electron with wave vector *k* in the α lead. And $H_{\mathrm{hyb}}$ captures the tunnel coupling between dots 1 and 3 and their respective leads:

(4) $$H_{\mathrm{hyb}}=\sum_{\alpha ki\mu }V_{\alpha ki\mu }{\hat{d}}_{i\mu }^{†}{\hat{c}}_{\alpha k\mu }+H.c.,$$

with $V_{\alpha ki\mu \mu }$ being the tunnel matrix element between *i*-th impurity and electrons with *k*-th state on the α -reservoir. For this paper, $V_{\alpha k\mu }$ is the electron tunneling strength between two leads. The effect of electron reservoirs on QDs is taken into account through the hybridization functions, $\Delta_{\mu v}\left(\omega \right)\equiv \Sigma_{\alpha }\Delta_{\alpha \mu \nu }\left(\omega \right)=\pi \Sigma_{\alpha k}V_{\alpha \mu k}V_{\alpha vk}^{*}\delta \left(\omega -\varepsilon_{\alpha k}\right)$, in the absence of applied chemical potentials. Generally, we adopt Lorentzian hybridization functions in the HEOM approach, that is, $\Delta_{\mu v}\left(\omega \right)=\delta_{\mu v}\Delta W^{2}/\left(\omega^{2}+W^{2}\right)$, with $\Delta =\Sigma_{\alpha }\Delta_{\alpha }$ being the overall dot-lead coupling strength and W is the bandwidth of the electrodes, W = 5 meV, and the reported results are not sensitive to the precise value of W within the parameter regime studied.

A magnetic flux ϕ threading the lead-free triangular loop modifies the inter-dot hopping amplitude through a phase factor. We use perturbation theory in the inter-dot tunneling *t*, treating it as small compared to the on-site Coulomb repulsion *U*. This approach allows us to derive an effective spin-exchange Hamiltonian from the microscopic quantum dot Hamiltonian $H_{\mathrm{dots}}$ [33,34] by integrating out high-energy charge excitations. The resulting Hamiltonian takes the form:

(5) $$\begin{array}{cc} H_{\mathrm{eff}\;}= & -t\left(1-n\right)\sum_{jk,\mu }\left({\hat{d}}_{j\mu }^{†}{\hat{d}}_{k\mu }+\;H.c.\;\right)\\ \; & +J\sum_{j<k}\left(\hat{S_{j}}\hat{S_{k}}-\frac{1}{4}{\hat{n}}_{j}{\hat{n}}_{k}\right)+\chi \hat{S_{1}}\left(\hat{S_{2}}\times \hat{S_{1}}\right), \end{array}$$

where *n* is the expectation value of the occupation number per dot, $n\equiv \frac{1}{3}\sum_{j=1}^{3}\langle {\hat{n}}_{j}\rangle$, and ${\hat{n}}_{j}={\hat{d}}_{j}^{†}{\hat{d}}_{j}$ is the number operator for electrons on dot *j*. $\hat{S_{1}}$, $\hat{S_{2}}$ and $\hat{S_{3}}$ are the spin operators on quantum dots, and $\hat{S_{j}}=\frac{1}{2}\sum_{\mu ,\mu^{\prime }}{\hat{d}}_{j\mu }^{†}\tau_{\mu \mu^{\prime }}{\hat{d}}_{j\mu^{\prime }}$, $\tau_{\mu \mu^{\prime }}$ are the Pauli matrices. The first term will vanish in the half-filling situation (n=1). The second term is the Heisenberg exchange interaction with $J=4t^{2}/U$. The third term is the chiral term with chiral operator [35] ${\hat{S}}_{1}\cdot \left({\hat{S}}_{2}\times {\hat{S}}_{3}\right)$, where χ is the chiral interaction with $\chi =24t^{3}\sin\left(2\pi \phi /\phi_{0}\right)/U^{2}$, and ϕ is the magnetic flux enclosed by the TTQD structure. Here, $\phi_{0}=hc/e$ is the unit of quantum flux. For simplicity, we let $\varphi =2\pi \phi /\phi_{0}$; thus, $\chi =24t^{3}\sin\left(\varphi \right)/U^{2}$.

The hierarchical equations of motion (HEOM) can accurately solve the three-impurity Anderson model [36]. Recent progress has substantially broadened HEOM’s scope and efficiency for quantum transport applications. Tanimura offered a comprehensive review of theoretical foundations and numerical implementations for both bosonic and fermionic systems, emphasizing its advantages over perturbative master equation approaches in strong-coupling regimes [37]. These developments establish HEOM as a reliable tool for exploring thermoelectric phenomena in multi-dot systems.

The HEOM framework derives from Feynman–Vernon influence functional path-integral theory [38] and employs Grassmann algebra for fermionic dissipation [39]. Consequently, HEOM provides a formally exact treatment of general open systems coupled to reservoir baths that satisfy Grassmann Gaussian statistics. The mathematical construction has been comprehensively discussed in previous references [40,41,42]. Here, we highlight key features particularly relevant to the quantum dot systems examined in this work’s main text.

The HEOM approach provides a universal framework for investigating quantum dot properties under equilibrium and non-equilibrium conditions through the reduced density operator, applicable to arbitrary system Hamiltonians. We present a concise derivation below. At time *t*, the reduced density operator of the system is defined as $\rho \left(t\right)={\mathrm{tr}}_{\mathrm{res}}\rho_{T}\left(t\right)$, where $\rho_{T}\left(t\right)$ denotes the total density operator of both system and reservoirs. The reduced density operator ρ(t) connects to its initial value at time $t_{0}$ through the reduced Liouville-space propagator $G\left(t,t_{0}\right)$:

(6) $$\rho \left(t\right)=G\left(t,t_{0}\right)\rho \left(t_{0}\right).$$

Using the Feynman–Vernon influence functional formalism, the path-integral representation of the reduced Liouville-space propagator takes the form

(7) $$G\left(\psi ,t;\psi_{0},t_{0}\right)=\int_{\psi_{0}\left[t_{0}\right]}^{\psi [t]}D\psi e^{iS[\psi ]}F\left[\psi \right]e^{-iS\left[\psi^{\prime }\right]},$$

where S[ψ] denotes the classical action of the reduced system and F[ψ] is the corresponding influence functional. Following the work of Zhenhua Li et al. [36], we invoke Wick’s theorem together with Grassmann algebra to express the influence functional F[ψ] as

(8) $$F\left[\psi \right]=\exp\left\{-\int_{0}^{t}\;d\tau R\left[\tau ,\left\{\psi \right\}\right]\right\},$$

where $R\left[\tau ,\left\{\psi \right\}\right]=\frac{i}{\hbar^{2}}\sum_{\alpha i\mu \sigma }A_{ij\mu }^{\bar{\sigma }}\left[\psi \left(t\right)\right]B_{\alpha i\mu }^{\sigma }\left[t,\psi \right]$, with σ=+,− and $\bar{\sigma }=-\sigma$. The Grassmann variables $A_{is}^{\bar{\sigma }}$ and $B_{\alpha i\mu }^{\sigma }$ are defined as

(9) $$A_{is}^{\bar{\sigma }}\left[\psi \left(t\right)\right]=d_{is}^{\sigma }\left[\psi \left(t\right)\right]+d_{is}^{\sigma }\left[\psi^{\prime }\left(t\right)\right],$$

(10) $$B_{\alpha i\mu }^{\sigma }\left[t,\psi \right]=-i\left[B_{\alpha i\mu }^{\sigma }\left(t,\psi \right)-B_{\alpha i\mu }^{\prime \sigma }\left(t,\psi^{\prime }\right)\right],$$

with

(11) $$\begin{array}{c} B_{\alpha i\mu }^{\sigma }\left(t,\psi \right)=\sum_{j}\int_{0}^{t}\;d\tau C_{\alpha ij\mu }^{\sigma }\left(t-\tau \right)d_{j\mu }^{\sigma }\left[\psi \left(\tau \right)\right],\\ B_{\alpha i\mu }^{\prime \sigma }\left(t,\psi^{\prime }\right)=\sum_{j}\int_{0}^{t}\;d\tau C_{\alpha ij\mu }^{\bar{\sigma }*}\left(t-\tau \right)d_{j\mu }^{\sigma }\left[\psi^{\prime }\left(\tau \right)\right]. \end{array}$$

Here, $C_{\alpha ij\mu }^{\sigma }\left(t\right)$ represents the reservoir correlation functions. In the present computational scheme, $C_{\alpha ij\mu }^{\sigma }\left(t\right)$ is decomposed into a sum of exponential terms by combining the fluctuation-dissipation theorem with the Cauchy residue theorem and the Padé spectral decomposition [43] of the Fermi function:

(12) $$C_{\alpha ij\mu }^{\sigma }\left(t\right)=\sum_{m=1}^{M}\eta_{\alpha ij\mu m}^{\sigma }e^{-\gamma_{\alpha ij\mu m}^{\sigma }t}.$$

The bath effects enter the equations of motion through *M* exponential terms. The auxiliary density operators (ADOs) $\left\{\rho_{j}^{n}=\rho_{j_{1}\ldots j_{n}}\right\}$ are determined by the time derivative of the influence functional. The composite index j≡(σμm) characterizes the transfer of an electron to or from (σ=+/−) the impurity state μ, while n=1,2,…,L specifies the truncation tier level. In the present study of the strong coupling system [36], setting L=4 is found sufficient for obtaining results that numerically converged within the standard accuracy of the HEOM treatment for this class of quantum-dot systems. The resulting hierarchy equations of motion can be written in the following compact form:

(13) $$\begin{array}{cc} {\dot{\rho }}_{j_{1}\ldots j_{n}}^{\left(n\right)}= & -\left(iL+\sum_{r=1}^{n}\gamma_{j}\right)\rho_{j_{1}\ldots j_{n}}^{\left(n\right)}-i\sum_{j}A_{j}\rho_{j_{1}\ldots j_{n}j}^{\left(n+1\right)}\\ \; & -i\sum_{r=1}^{n}{\left(-\right)}^{n-r}C_{j_{r}}\rho_{j_{1}\ldots j_{r-1}j_{r+1}\ldots j_{n}}^{\left(n-1\right)}. \end{array}$$

In the above, L is the Liouvillian superoperator of the quantum dots, which incorporates electron-electron interactions, and is defined as $L\cdot \equiv \left[H_{\mathrm{dot}},\cdot \right]$. The Grassmannian superoperators $A_{\bar{j}}\equiv A_{is}^{\bar{\sigma }}$ and $C_{j}\equiv C_{ijsm}^{\sigma }$ act on an arbitrary operator $\hat{O}$ according to $A_{j}\hat{O}\equiv \left[{\hat{d}}_{is}^{\hat{\sigma }},\hat{O}\right]$ and $C_{j}\hat{O}\equiv \eta_{j}{\hat{d}}_{is}^{\sigma }\hat{O}+\eta_{j}^{*}\hat{O}{\hat{d}}_{is}^{\sigma }$, respectively.

Furthermore, only the first-tier auxiliary density operators are required to accurately evaluate the transient current flowing through electrode α:

(14) $$I_{\alpha }\left(t\right)=i\sum_{i\mu }{\mathrm{tr}}_{\mathrm{sys}}\left[\rho_{\alpha i\mu }^{†}\left(t\right){\hat{d}}_{i\mu }-{\hat{d}}_{i\mu }^{†}\rho_{\alpha i\mu }^{-}\left(t\right)\right].$$

To confirm that the reported current corresponds to a true stationary value, we examined the time evolution of the transport current I(t) within the HEOM framework. Starting from the initial state, the current exhibits a transient response after the bias is applied, then gradually approaches a time-independent limit as the reduced density matrix and the auxiliary density operators converge. In the calculations presented in this work, the transient part decays within the simulation time window, and I(t) reaches a stable plateau without visible long-term drift. The stationary current reported in the manuscript is therefore defined as the long-time limit,

$$I_{\mathrm{st}}=\lim_{t\to \infty }I\left(t\right),$$

taken after the convergence of the full HEOM dynamics. This behavior confirms that the numerical results correspond to a nonequilibrium steady state rather than to a transient transport regime.

In addition, the spectral function is obtained by propagating the correlation functions $C_{a_{i\mu }a_{i\mu }^{†}}\left(t\right)$ and $C_{a_{i\mu }^{†}a_{i\mu }}\left(t\right)$ in time and subsequently performing a semi-infinite Fourier transform. This procedure directly yields the spectral function $A_{i\mu }\left(\omega \right)$ associated with spin μ in the *i*-th quantum dot. The fluctuation-dissipation theorem further allows the spectral density function of the quantum dots to be extracted from these correlation functions. The spectral function is given by

(15) Aiμ(ω)=1πRe∫0∞dtCaiμaiμ†(t)+Caiμ†aiμ(t)*e−iωt.

## 3. Results and Discussion

Figure 1 compares the transport current as a function of *U* for the triangular triple quantum dots (TTQDs) and linear triple quantum dots (LTQDs), with all other parameters held identical and the interdot hopping fixed at t=0.25 meV. For the LTQD chain, the transport current decreases monotonically as *U* increases from 2t to 5t. This monotonic suppression is consistent with the standard expectation from the Anderson impurity model in the strong-coupling regime. In contrast, the triangular triple quantum dot system exhibits a pronounced non-monotonic dependence of the transport current on *U*. Starting from U=2t, the current first increases with *U*, reaches a maximum at approximately U=3.5t, and subsequently decreases for larger values of *U*. This non-monotonic behavior represents the central finding of this work and signals the presence of a mechanism that, over a certain range of Coulomb interaction, enhances the transport current despite the growing on-site repulsion.

**Figure 2:**
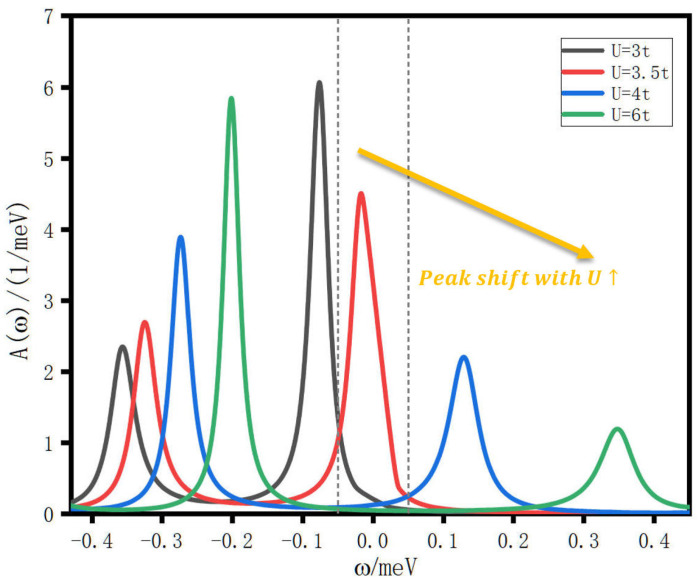
Spectral function A(ω) of the triangular triple quantum dot system in the vicinity of the Fermi level ω=0, computed at a representative on-site Coulomb interaction *U* selected from both the rising and the falling portions of the current curve in Figure 1.

To elucidate the mechanism underlying the non-monotonic current, we compute the frequency-resolved spectral function A(ω) at several representative values of *U* along both the ascending and descending portions of the current curve, as shown in Figure 2. Within the small-bias regime, the current is predominantly controlled by the spectral weight available inside the conducting window around the Fermi level, which is shown by the dotted line in Figure 2. For clarity, we restrict our presentation to the spectral weight in the vicinity of the Fermi level ω=0.

At the smallest value of *U*, both spectral peaks reside below the Fermi level. As *U* increases, the peak closer to the Fermi level shifts upward, while its spectral weight decreases. The other peak gains weight but remains farther from ω=0. This redistribution of spectral weight between the two peaks is a hallmark of correlation-driven restructuring of the electronic spectrum. In the presence of Coulomb interactions, the eigenstates of the system are no longer simple single-particle states but rather correlated many-body states that incorporate the effects of electron-electron repulsion. The energy of these states depends on both the kinetic energy associated with interdot hopping and the potential energy arising from Coulomb repulsion. As *U* increases, states with lower total Coulomb energy become relatively more favorable, and the system reorganizes its spectral weight to reflect this energetic preference. In the triangular geometry, certain many-body states correspond to charge configurations where electrons are distributed to minimize mutual repulsion while maintaining connectivity to the leads. These states can have energies that increase less rapidly with *U* than other states, or in some cases, their energies relative to the Fermi level may even decrease due to the specific form of the interaction Hamiltonian in the triangular topology. This differential energy shift causes spectral peaks to move toward the Fermi level, enhancing transport when they enter the conducting window.

The non-monotonic behavior of the current is directly explained by the trajectory of the right spectral peak relative to the bias window centered at the Fermi level. At U=3t, the right peak has shifted to a position near ω=−0.08t, while the left peak resides at ω=−0.35t. The right peak is now in close proximity to the Fermi level, and its spectral weight, although reduced compared to its value at U=2t, still contributes significantly to transport. As *U* continues to increase and reaches U=3.5t, the right peak arrives in the immediate vicinity of the Fermi level. At this point, the left peak resides at ω=−0.31t, having also shifted toward the Fermi level compared to its earlier position, but remaining well outside the narrow conducting window defined by the small applied bias. The transport current attains its maximum value at U=3.5t precisely because the antibonding spectral resonance is optimally aligned with the bias window. Physically, the antibonding spectral resonance, driven toward the Fermi level by the increasing Coulomb repulsion, sweeps through the narrow conducting window. The alignment of this resonance with the Fermi level maximizes the transmission probability and, consequently, the current. For *U* larger than 3.5t, the right peak continues to shift and eventually crosses the Fermi level, moving out of the conducting window on the opposite side. At the same time, the ongoing suppression of the spectral weight of this peak by the growing Coulomb repulsion further reduces its contribution to the current. The combined effect of the peak moving away from the optimal position and losing spectral weight produces the observed decrease in the transport current at large *U*. Although the left peak continues to slowly approach the Fermi level, it never enters the bias window, and its growing spectral weight cannot compensate for the loss of the right peak’s contribution. The physical mechanism driving this energy shift involves the interplay between Coulomb repulsion and the quantum interference inherent to the triangular geometry. In the triangular configuration, electrons can traverse between the two connected dots either directly through interdot hopping or indirectly via the unconnected third dot. These two pathways interfere, and the relative phase between them depends on the energy spectrum of the system. Coulomb interactions modify the energy levels of many-body states, thereby altering the interference conditions. In systems with quantum interference, the position of transmission resonances depends sensitively on the phase relationships between different transport pathways. Coulomb interactions introduce energy-dependent phase shifts that can tune these resonances into alignment with the Fermi level. This tuning effect is absent in linear geometries, where the lack of alternative pathways prevents the formation of interference-based resonances that can be manipulated by interactions.

**Figure 3:**
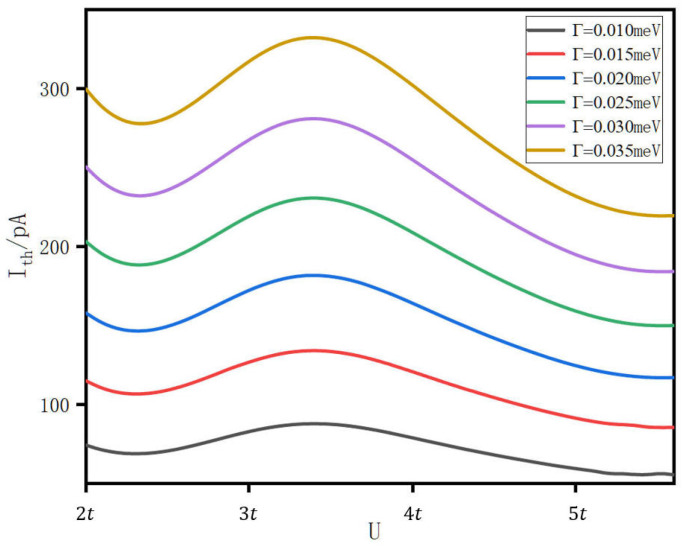
Transport current *I* through the TTQDS as a function of the on-site Coulomb interaction *U*, computed for several values of the lead-dot coupling strength Γ.

To assess the robustness of this interpretation, we present in Figure 3 the transport current as a function of *U* for several values of the dot-electrode coupling strength Γ.

Increasing Γ systematically raises the magnitude of the transport current across the entire range of *U*, which is physically expected; a larger Γ broadens the quasiparticle resonances and increases the tunneling rate, thereby enhancing the current for any given alignment between the spectral peak and the transport window. The qualitative non-monotonic structure of the *I*-*U* curve is, however, preserved for all values of Γ examined. The position of the current maximum shifts only weakly with Γ, consistent with the fact that the peak migration in energy is controlled by the many-body self-energy and is therefore primarily a function of U/t rather than of Γ.

Reducing Γ diminishes the current amplitude and sharpens the spectral features, but again leaves the essential *U*-dependence unchanged. This insensitivity of the current’s qualitative behavior to Γ is an important consistency check: it confirms that the non-monotonic current is not an artifact of a particular coupling regime but is a robust property of the triangular topology and the interplay between Coulomb interaction and the closed-loop geometry. The spectral function analysis of Figure 2 and the coupling-strength sweep of Figure 3 together provide a consistent account of the anomalous current enhancement. The triangular geometry generates chiral quasiparticle states whose energies are pulled toward the Fermi level by the Coulomb interaction; as these states pass through the transport window, the current rises and then falls. This behavior persists across a wide range of Γ, confirming that it reflects the topology of the system rather than any fine-tuning of parameters.

**Figure 4:**
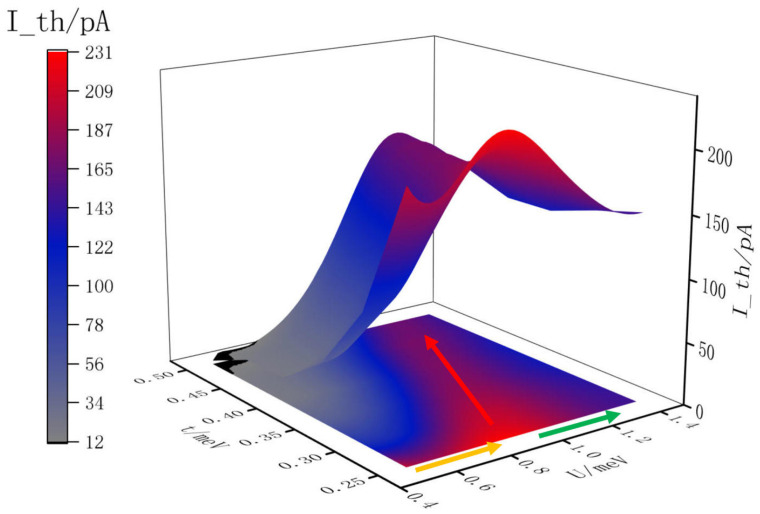
Three-dimensional representation of transport current as a function of interdot hopping amplitude t and on-site Coulomb repulsion U for the triangular triple quantum dot system. Yellow arrows indicate regions where transport current increases with growing U, demonstrating the counterintuitive enhancement effect. Green arrows mark regions where further increases in U suppress the current due to Coulomb blockade effects. Red arrows highlight the trend that larger values of t require correspondingly larger values of U to achieve the maximum transport current, revealing that the phenomenon is governed by the dimensionless ratio U over t. The plot demonstrates that interaction-enhanced transport occurs in an intermediate regime where U and t are comparable in magnitude.

To further examine this effect, we compute the transport current $I_{\mathrm{th}}$ as a function of both *U* and *t* over a wide range of parameters. The results are summarized in Figure 4, which presents a three-dimensional plot of the threshold current $I_{\mathrm{th}}$ in the *t*-*U* plane. The three-dimensional surface shows a nontrivial structure. The yellow arrow in the figure traces a path of fixed *t* along which *U* increases, corresponding to the direction of current enhancement described in the previous section. Following the yellow arrow, the current rises from its value at small *U*, reaches a maximum, and then falls along the trajectory indicated by the blue arrow. The non-monotonic *U*-dependence of the current at fixed *t* is thus consistently observed across the full range of *t* values explored, confirming that the phenomenon is not limited to a specific choice of hopping amplitude but is a generic feature of the TTQD geometry.

The red arrow in Figure 4 reveals a systematic trend of particular physical significance. As *t* increases, the value of *U* at which the current reaches its maximum also increases. This means that the current peak shifts to larger *U* as the hopping amplitude grows. The enhancement stage typically occurs when U is between 2t and 3t. The implication is that the anomalous current enhancement is a phenomenon governed by the interplay between *t* and *U* through their ratio, rather than by either parameter independently. This phenomenon has an important physical corollary. It means that the current enhancement is not a perturbative effect that vanishes at small U/t, nor is it a strong-coupling phenomenon that only appears at U≫t. Instead, it occurs in an intermediate-coupling regime where both the kinetic and interaction energy scales are comparable, and where quantum interference in the triangular loop and Coulomb-driven renormalization of the chiral-state energies cooperate to produce the anomalous transport signature. The linear geometry, lacking the loop structure, cannot support the closed-loop virtual processes that drive orbital renormalization and therefore shows no such enhancement regardless of *U* and *t*.

## 4. Summary and Conclusions

In this work, we studied transport through a triangular triple-quantum-dot system. The main result is that the current shows a non-monotonic dependence on the on-site Coulomb interaction *U*. Under the same conditions, the linear triple quantum dot shows the expected monotonic decrease of current with increasing *U*, whereas the triangular system shows a current enhancement at intermediate *U* followed by suppression at larger *U*. The spectral analysis shows that this behavior comes from an interaction-driven shift of a resonance toward the Fermi level. When this resonance enters the bias window, the current increases; when it moves out of the bias window and loses spectral weight, the current decreases. Finally, by mapping the transport current across a wide range of both *U* and *t*, we have demonstrated that the current enhancement is governed not by either parameter alone but by the combined effect of *U* and *t*. More detailed optimization and a finer mapping of the relevant parameter range are part of our future research. These results suggest that the triangular quantum dot geometry may serve as a useful experimental platform for engineering interaction-tunable transport responses, in which the current through the device can be increased rather than suppressed by strengthening the on-site Coulomb repulsion, provided the hopping amplitude and interaction strength are tuned to the appropriate intermediate-coupling regime.

The principle identified in this work is not limited to the specific triangular three-dot geometry. More complex loop-containing networks, including tetrahedral four-dot clusters, ladder geometries, and two-dimensional arrays with embedded loops, while maintaining the symmetry of particle-hole pairs, are all expected to exhibit analogous interaction-driven spectral renormalization effects arising from the closed-loop virtual hopping processes identified here. As network size and connectivity increase, the number of independent chiral channels grows, and the interplay among frustration, interactions, and interference is expected to give rise to increasingly rich transport phenomenology. The present work provides both the conceptual framework and the specific spectral mechanism needed to analyze and interpret these more complex cases, and it motivates a systematic study of how the topology of the dot network determines the sign and magnitude of the interaction-driven current correction across a broader class of correlated nanoscale conductors.

## Data Availability

The data that support the findings of this study are available from the corresponding author upon reasonable request.
